# Effects of the COVID-19 Pandemic on Obsessive-Compulsive Symptoms Among University Students: Prospective Cohort Survey Study

**DOI:** 10.2196/21915

**Published:** 2020-09-30

**Authors:** Guangjun Ji, Wenjun Wei, Kai-Chen Yue, Heng Li, Li-Jing Shi, Jian-Dong Ma, Chen-Yang He, Sheng-Sheng Zhou, Zongya Zhao, Tao Lou, Jie Cheng, Shi-Chang Yang, Xian-Zhang Hu

**Affiliations:** 1 Xinxiang Medical University Xinxiang China; 2 The Second Affiliated Hospital of Xinxiang Medical University Xinxiang City China

**Keywords:** COVID-19, fear, anxiety, obsessive-compulsive disorder, OCD, Yale-Brown Obsessive-Compulsive Scale, university student, mental health

## Abstract

**Background:**

The COVID-19 pandemic is associated with common mental health problems. However, evidence for the association between fear of COVID-19 and obsessive-compulsive disorder (OCD) is limited.

**Objective:**

This study aimed to examine if fear of negative events affects Yale-Brown Obsessive-Compulsive Scale (Y-BOCS) scores in the context of a COVID-19–fear-invoking environment.

**Methods:**

All participants were medical university students and voluntarily completed three surveys via smartphone or computer. Survey 1 was conducted on February 8, 2020, following a 2-week-long quarantine period without classes; survey 2 was conducted on March 25, 2020, when participants had been taking online courses for 2 weeks; and survey 3 was conducted on April 28, 2020, when no new cases had been reported for 2 weeks. The surveys comprised the Y-BOCS and the Zung Self-Rating Anxiety Scale (SAS); additional items included questions on demographics (age, gender, only child vs siblings, enrollment year, major), knowledge of COVID-19, and level of fear pertaining to COVID-19.

**Results:**

In survey 1, 11.3% of participants (1519/13,478) scored ≥16 on the Y-BOCS (defined as possible OCD). In surveys 2 and 3, 3.6% (305/8162) and 3.5% (305/8511) of participants had scores indicative of possible OCD, respectively. The Y-BOCS score, anxiety level, quarantine level, and intensity of fear were significantly lower at surveys 2 and 3 than at survey 1 (*P*<.001 for all). Compared to those with a lower Y-BOCS score (<16), participants with possible OCD expressed greater intensity of fear and had higher SAS standard scores (*P*<.001). The regression linear analysis indicated that intensity of fear was positively correlated to the rate of possible OCD and the average total scores for the Y-BOCS in each survey (*P*<.001 for all). Multiple regressions showed that those with a higher intensity of fear, a higher anxiety level, of male gender, with sibling(s), and majoring in a nonmedicine discipline had a greater chance of having a higher Y-BOCS score in all surveys. These results were redemonstrated in the 5827 participants who completed both surveys 1 and 2 and in the 4006 participants who completed all three surveys. Furthermore, in matched participants, the Y-BOCS score was negatively correlated to changes in intensity of fear (*r*=0.74 for survey 2, *P*<.001; *r*=0.63 for survey 3, *P*=.006).

**Conclusions:**

Our findings indicate that fear of COVID-19 was associated with a greater Y-BOCS score, suggesting that an environment (COVID-19 pandemic) × psychology (fear and/or anxiety) interaction might be involved in OCD and that a fear of negative events might play a role in the etiology of OCD.

## Introduction

At the end of 2019, an outbreak of COVID-19, caused by the novel coronavirus SARS-CoV-2 was reported in Wuhan, China [1]. With the growing number of cases and deaths, fear and uncertainty have spread around the globe as COVID-19 continues to capture the world’s attention. Since the end of January 2020, many provinces in China, including Henan Province, implemented quarantine measures, which may further instill fear of the virus in communities. At the same time, public education on disease prevention and environmental hygiene was emphasized across the country. Information outlets, such as the internet, television, radio, newspapers, cellphones, and social media (eg, WeChat), were used to disseminate advice on how to prevent infection (eg, stay at home, wear face masks, wash hands frequently, and/or sanitize hands). In addition, the rapid transmission of COVID-19, its approximately 2% fatality rate, lack of effective treatments and vaccines, and mass quarantine measures are associated with common mental health problems (eg, fear, anxiety, depression, and sleep problems) in subpopulations, including COVID-19 patients, those with close contact to infectees, the public, and health care professionals [2,3]. A study including 1210 respondents from 194 cities in China found that 54% of respondents rated the psychological impact of the COVID-19 outbreak as moderate or severe; 29% reported moderate-to-severe anxiety symptoms; and 17% reported moderate-to-severe depressive symptoms [4]. However, there is a lack of research on the effects of the COVID-19 pandemic on specific mental disorders, such as obsessive-compulsive disorder (OCD).

OCD is a chronic and debilitating mental disorder and tends to be treatment refractory. It is characterized by unwanted intrusive thoughts (obsessions) and repetitive compulsive behaviors or mental rituals (compulsions). Individuals perform compulsions in response to the distress associated with the content of the obsessions. Often of early onset (ie, before 18 years of age), OCD impacts 2% to 3% of the US population [5] and affects individuals throughout their lives, leading to a diminished quality of life for patients and their families, reduced productivity, and high health care costs. OCD accounts for 2.2% of total years lived with disability, which is approximately the same percentage as schizophrenia [6].

OCD has been reported to have several specific clinical characteristics. First, individuals manifest OCD symptoms only under certain situations that usually invoke a fear of negative events. Second, more than 90% of the general population has experienced intrusive thoughts [7]. Third, the more effort put into controlling the obsession, the more frequently and intensely it intruded the patient’s mind [8]. Forth, compulsions can make intrusive thoughts become more frequent, repetitive, and disturbing [9]. Fifth, the performance of repetitive behaviors (eg, handwashing or checking) are generally related to a fear of negative events, such as fear of contamination or fear of a house catching on fire. Finally, in an OCD symptom–induced situation, the fear of negative events, obsessions, and compulsions can be considered as stressors and their effects on individuals can be neutralized when appropriate coping strategies are used [10-12]. This evidence suggests that fear of negative events is involved in symptom development and maintenance. For example, individuals with OCD repetitively check—for instance, the door or stove—due to a fear of loss of property, and spend a long time on handwashing due to a fear of contamination [13,14]. In addition, worry, disgust, and “just-not-right” sentiments can be involved in the onset of OCD [15,16]. Recently, a study in pediatric OCD found that individuals with OCD exhibit greater fear acquisition and impaired inhibitory learning compared to healthy controls [17]. However, there is lack of prospective studies on the relationship between the onset of OCD and a fear of negative events [18].

In this prospective study, we conducted surveys on students of the Xinxiang Medical University (XXMU) at three timepoints during the COVID-19 pandemic. We primarily sought to investigate whether fear of COVID-19 affects the prevalence of possible OCD based on a score of ≥16 on the Yale-Brown Obsessive-Compulsive Scale (Y-BOCS). We also aimed to investigate the predictors for possible OCD. We hypothesized that fear of COVID-19 infection would be correlated to Y-BOCS scores.

## Methods

### Participants and Procedure

In this prospective cohort study, we surveyed college students at XXMU, including medical and nonmedical students. All participants voluntarily completed the survey via smartphone or computer at three timepoints.

The initial survey (survey 1) was distributed on February 8, 2020, when the participants were on winter break under a high level quarantine due to increases in COVID-19 cases reported. Survey 2 was distributed on March 15, 2020, when the participants had been taking online courses for 2 weeks under a moderate level of quarantine. Survey 3 was distributed on April 30, 2020, when the participants were still taking academic courses at home under a low quarantine level with no new cases reported.

**Figure 1:**
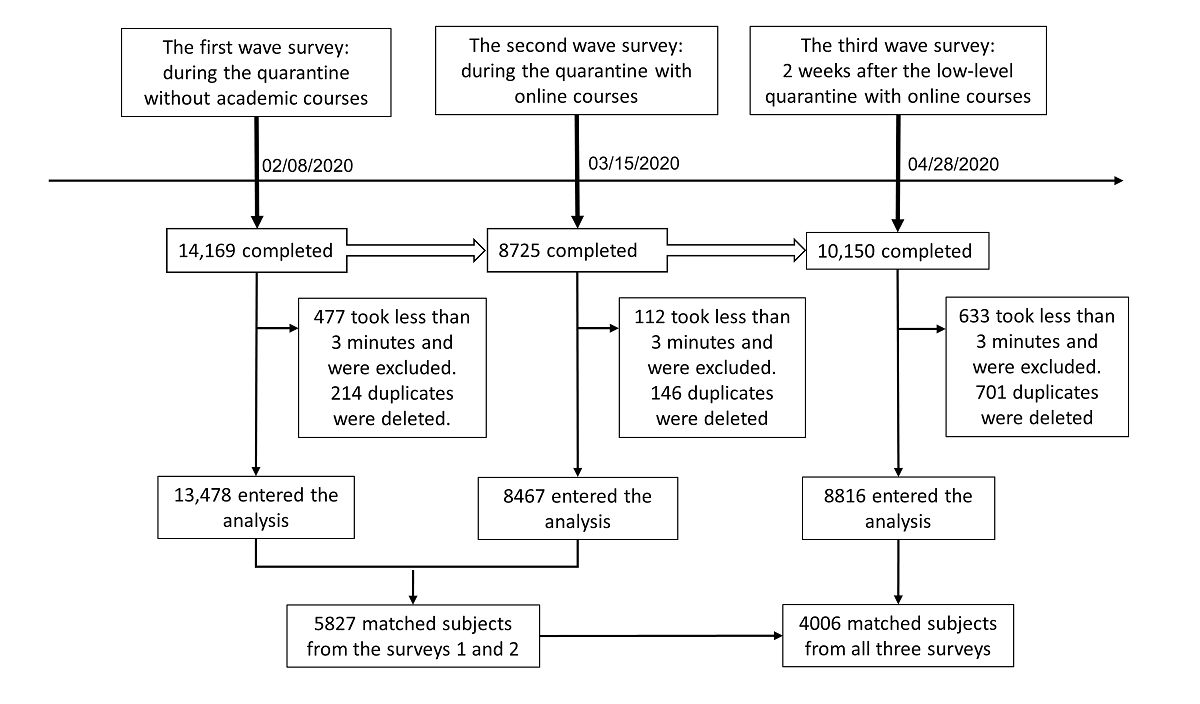
Flow diagram of surveys among university students.

For survey 1, we received 14,691 completed questionnaires. Among them, the 477 students who completed the survey in less than 3 minutes were excluded, and 214 duplications were deleted. For survey 2, we received 8725 completed questionnaires. Among them, the 112 students who completed the survey in less than 3 minutes were excluded, and 146 duplications were deleted. For survey 3, we received 10,150 completed questionnaires. Among them, the 633 students who completed the survey in less than 3 minutes were excluded, and 701 duplications were deleted. In order to track the identity of individuals who completed all three surveys, we used nicknames, age, gender, address (city), grade, and major as ID variables; there were 5827 ID-matched participants from surveys 1 and 2 and 4006 ID-matched participants from all three surveys, after excluding one ID duplicate in each survey and those who completed the surveys in 3 minutes (Figure 1). In the surveys, quarantine level in different areas where participants lived were announced by the government and were designated as low (score of 1), medium (score of 2), and high (score of 3), which reflected the severity of the COVID-19 pandemic in that area.

The survey protocol was approved by the Committee on Human Research at XXMU. Since the study involved internet technologies, this ensured compliance with the principles of voluntary participation. All participants provided electronic informed consent.

### Assessment

We used a battery of questionnaires in our surveys, including questions on basic information (age, gender, only child vs sibling[s], enrollment year, major), knowledge on COVID-19 (0 for “do not know” to 3 for “very knowledgeable”), level of fear (0 for “no fear” to 9 for “extreme fear”), as well as the Y-BOCS and the SAS. The Y-BOCS is an undisputed gold standard to evaluate the severity of OCD symptoms [19,20]. It is the most widely used semistructural scale in both clinical and research settings. It consists of a comprehensive symptom checklist to identify the specific type and content of obsessive and compulsive symptoms in addition to a 10-item rating scale. After inquiring about what types of obsessions and compulsions the patient experiences using a standard checklist, individuals are asked to identify their main symptoms (obsessions and compulsions) and respond to a series of questions. The scale is divided into two subscales that separately measure obsessions and compulsions. For each subscale, five aspects of obsessive and compulsive pathology are each rated on a scale ranging from 0 (no symptoms) to 4 (extreme symptoms): time spent, degree of interference, distress, resistance (greater resistance is assigned lower scores), and perceived control over the symptom. Subscale scores are summed to yield a Y-BOCS total score. There is a moderate correlation in consistency and discrepancy between self-reported and clinician-rated Y-BOCS scores; the highest correlation is observed for the compulsion subscale and patients tend to rate symptoms lower than clinicians [21,22]. Since many studies use a Y-BOCS score ≥16 as an inclusion criteria for OCD, we defined that score as “possible OCD” in this study. We used the SAS to quantify a participant's level of anxiety [23]. The SAS is a 20-item self-report assessment device built to measure anxiety levels, based on scoring in 4 groups of manifestations: cognitive, autonomic, motor, and central nervous system symptoms. Responding to each item, a person should indicate how much each statement applies to him or her in the past 1 or 2 weeks. Each question is scored on a scale of 1 to 4 (1: “a little of the time,” 2: “some of the time,” 3: “a good part of the time,” 4: “most of the time”). Of the 20 items, five items are negatively worded to avoid the problem of set response (ie, careless responding). The total raw scores range from 20 to 80. The raw score is used as an anxiety-severity score. The total score of 20-44 indicates normal range, 45-59 indicates mild-to-moderate anxiety, 60-74 indicates marked-to-severe anxiety, and >75 indicates extreme anxiety.

### Statistical Analyses

The microdata from the three surveys of the same population were analyzed using SPSS, version 24 (IBM Corp). Demographic characteristics of participants were tabulated using means and standard deviations for continuous variables and frequency distributions for categorical variables. The repeated measure analyses (Wilks lambda) were performed for the 5827 participants who were matched using ID variables from the first two surveys and for the 4006 matched participants from all three surveys, to examine the changes in Y-BOCS score, anxiety levels (SAS score), and intensity of fear of COVID-19. For all eligible participants from all surveys, analysis of variance (ANOVA), chi-square tests, and regression analyses were performed to examine demographic characteristics and Y-BOCS score predictors.

## Results

There were 13,478 participants in survey 1, 8467 participants in survey 2, and 8816 participants in survey 3 who were included in the analysis. The participants were aged between 17-50 years (mean 21.3, SD 2.5 years for survey 1; mean 21.2, SD 2.3 years for survey 2; mean 20.9, SD 2.0 years for survey 3; this mean age was lower than those of surveys 1 and 2, *P*<.001). In total, 664 (4.9%) participants in survey 1, 274 (3.2%) participants in survey 2, and 199 (2.6%) participants in survey 3 were aged ≥26 years old. The proportion of the participants who majored in clinical medicine was higher in survey 1, compared to those in surveys 2 and 3 (*P*<.001). There were 5827 participants who had at least five out of six ID variables matched in surveys 1 and 2, and 4006 participants were matched across all three surveys. The gender composition ratio and the rate of having one or more sibling(s) were not significantly different across survey participants. The distribution of participants in terms of enrollment year (2015-2019) was different between the three surveys (χ^2^_4_=151.6, *P*<.001) (Table 1).

**Table 1:** Demographic characteristics and questionnaire score.

Characteristic		Survey 1	Survey 2	Survey 3	F or χ^2^	*P* value
Age (year), mean (SD)		21.3 (2.5)	21.2 (2.3)	20.9 (2.0)	F=85.6	<.001
Intensity of fear, mean (SD)		7.8 (2.0)	6.7 (2.2)	6.5 (2.2)	F=1147.9	<.001
Y-BOCS^a^ score, mean (SD)		7.9 (5.7)	4.8 (5.1)	4.5 (5.1)	F=1366.6	<.001
SAS^b^ standard score, mean (SD)		36.9 (7.9)	36.1 (8.2)	36.2 (8.1)	F=34.2	<.001
Quarantine level, mean (SD)		2.44 (0.7)	1.26 (0.5)	1.00 (0.0)	F=27,129.4	<.001
**Age (years), n (%)**					χ^2^=113.6	<.001
	<26	12,814 (95.1)	8193 (96.7)	8617 (97.4)		
	≥26	664 (4.9)	274 (3.3)	199 (2.6)		
**Gender, n (%)**					χ^2^=1.8	.41
	Male	4662 (34.6)	2991 (35.3)	3113 (35.3)		
	Female	8816 (65.4)	5476 (64.7)	5703 (54.7)		
**Major, n (%)**					χ^2^=227.3	<.001
	Clinical	8549 (63.4)	4576 (54.0)	5259 (59.7)		
	Basic medical	3428 (26.4)	2902 (34.3)	2467 (28.0)		
	Nonmedical	1501 (11.1)	989 (11.7)	1090 (12.3)		
**Sibling(s), n (%)**					χ^2^=1.5	.48
	No	2452 (18.2)	1495 (17.7)	1557 (17.7)		
	Yes	11,026 (81.8)	6972 (82.3)	7259 (82.3)		
**Year of enrollment, n (%)**					χ^2^=360.9	<.001
	2015	1319 (10.0)	941 (11.1)	665 (7.5)		
	2016	2343 (17.7)	1863 (22.0)	1403 (15.9)		
	2017	2997 (22.6)	1511 (17.8)	2013 (22.8)		
	2018	3274 (24.7)	2169 (25.6)	2474 (28.1)		
	2019	3017 (22.8)	1883 (22.2)	2206 (25.0)		
	Other	291 (2.2)	92 (1.3)	55 (0.6)		
**Y-BOCS score^c^, n (%)**					χ^2^=704.5	<.001
	≥16	1519 (11.3)	305 (3.6)	305 (3.5)		
	<16	11,959 (88.7)	8162 (96.4)	8511 (96.5)		

^a^Y-BOCS: Yale-Brown Obsessive-Compulsive Scale.

^b^SAS: Zung Self-Rating Anxiety Scale.

^c^The odds ratio for the Y-BOCS was 2.4 (95% CI 2.2-2.7).

In survey 1, 11.3% (n=1519) of participants had a Y-BOCS score ≥16 (possible OCD); this was significantly higher than the 3.6% (n=305) observed in survey 2 (χ^2^_1_=401.2, odds ratio [OR] 2.4, 95% CI 2.2-2.7, *P*<.001) and 3.5% (n=305) in survey 3 (χ^2^_1_=431.9, OR 3.5, 95% CI 3.1-4.0, *P*<.001). Compared to the baseline, the self-reported intensity of fear of COVID-19, Y-BOCS score, SAS standard score, and quarantine level (1=low, 2=medium, and 3=high) were significantly reduced among surveys 2 and 3 participants (*P*<.001 for all) (Table 1).

In the 5827 matched participants from surveys 1 and 2 and the 4006 matched participants from all three surveys, the repeated measure analysis (Wilks lambda) showed that the Y-BOCS score, the SAS standard score, the intensity of fear of COVID-19, and the quarantine level of surveys 2 and 3 decreased significantly from baseline (*P*<.001 for all). The Y-BOCS score, intensity of fear of COVID-19, and quarantine level were lower in survey 3 than in survey 2, while the SAS standard score in survey 3 was higher than that in survey 2 (*P*<.001 for all) (Table 2).

**Table 2:** Repeated measure analysis (Wilks lambda) in matched samples between surveys 1 and 2 (n=5827) and across all three surveys (n=4006).

Variable		Survey 1	Survey 2	Survey 3	F (*df*)	*P* value
**Surveys 1 and 2**						
	Y-BOCS^a^ score, mean (SD)	8.0 (5.6)	4.7 (4.9)	—^b^	1858.6 (1)	<.001
	SAS^c^ score, mean (SD)	36.6 (7.6)	35.7 (7.9)	—	81.2 (1)	<.001
	Intensity of fear, mean (SD)	7.8 (2.0)	6.6 (2.3)	—	1357.9 (1)	<.001
	Quarantine level, mean (SD)	2.5 (0.7)	1.3 (0.5)	—	21,371.4 (1)	<.001
**All three surveys**						
	Y-BOCS score, mean (SD)	7.9 (5.5)	4.7 (4.9)	4.3 (4.9)	823.8 (2)	<.001
	SAS score, mean (SD)	36.3 (7.4)	35.2 (7.6)	35.6 (7.9)	41.2 (2)	<.001
	Intensity of fear, mean (SD)	7.7 (2.0)	6.6 (2.2)	6.4 (2.2)	707.2 (2)	<.001
	Quarantine level, mean (SD)	2.5 (0.7)	1.3 (0.4)	1.0 (0.03)	9627.4 (2)	<.001

^a^Y-BOCS: Yale-Brown Obsessive-Compulsive Scale.

^b^Not applicable.

^c^SAS: Zung Self-Rating Anxiety Scale.

To further analyze the characteristic of participants, the two-way ANOVA analysis using two independent variables of the surveys and Y-BOCS score (dichotomously grouped into “possible OCD” with a score ≥16 and <16) was performed. Significant differences of Y-BOCS score, intensity of fear, SAS standard score, and quarantine level were found among the groups (*P*<.001), in which no statistical difference in Y-BOCS score was found between the participants with possible OCD across all three surveys. In surveys 2 and 3, no difference was found in quarantine level between the participants with possible OCD and those with a Y-BOCS score <16 (Table 3). In addition, the chi-square test was applied to test the distribution of possible OCD in the survey participants. The prevalence of possible OCD in males was higher than that in female across all surveys. Taking age into account, the rates of possible OCD in males aged <26 years were higher than those in females (*P*=.001, *P*=.002, and *P*<.001 for surveys 1, 2, and 3, respectively), while the rates of possible OCD were not significantly different between males and females aged ≥26 years across all three surveys. The distribution of possible OCD was significantly different in terms of intensity of fear (*P*≤.001). The rate of possible OCD in participants who had sibling(s) was higher than that in those who had no sibling(s) (χ^2^_1_=11.2, *P*=.001) in survey 1, but no difference was found in surveys 2 and 3. The distribution of possible OCD in terms of the year of enrollment was different in surveys 2 and 3 (*P*=.03 and *P*=.02; Table 3).

**Table 3:** Comparison of participants with higher Yale-Brown Obsessive-Compulsive Scale (Y-BOCS) scores (≥16) to those with lower Y-BOCS scores (<16) across all three surveys.

Variable			Survey 1		Survey 2		Survey 3	
			Y-BOCS score <16	Y-BOCS score ≥16	Y-BOCS score <16	Y-BOCS score ≥16	Y-BOCS score <16	Y-BOCS score ≥16
Age (years), mean (SD)			21.3 (2.5)	21.4 (2.4)	21.2 (2.3)	21.2 (2.1	20.9 (2.0)	21.0 (1.9)
Y-BOCS score, mean (SD)			6.5 (4.3)	19.0 (3.2)^a^	4.3 (4.3)	19.0) (3.73^a^	4.0 (4.2)	19.3 (2.9)^a^
Intensity of fear, mean (SD)			7.7 (2.0)	8.7 (1.7)^a^	6.6 (2.3)	7.3 (2.2)^a^	6.5 (2.2)	7.3 (2.0)^a^
SAS standard score, mean (SD)			36.0 (7.1)	44.6 (9.4)^a^	35.6 (7.5)	50.1 (12.6)^a^	35.7 (7.5)	50.5 (10.6)^a^
Quarantine level, mean (SD)			2.4 (0.7)	2.5 (0.6)^b^	1.3 (0.5)	1.3 (0.4)	1.0 (0.0)	1.0 (0.0)
**Gender, n (%)**								
	Male		4081 (87.5)	581 (12.5)^b^	2860 (95.6)	131 (4.4)^b^	2974 (95.5)	139 (4.5)^a^
	Female		7878 (89.4)	938 (10.6)	5302 (97.3)	174 (2.7)	5537 (97.1)	166 (2.9)
**Age, n (%)**								
	**<26**							
		Male	3865 (87.4)	556 (12.6)^b^	2772 (95.6)	129 (4.4)^b^	2911 (95.5)	137 (4.5)^a^
		Female	7499 (89.3)	894 (10.7)	5127 (96.9)	165 (3.1)	5407 (97.1)	162 (2.9)
	**≥26**							
		Male	216 (89.6)	25 (10.4)	91 (97.8)	2 (2.2)	63 (96.9)	2 (3.1)
		Female	379 (89.6)	44 (10.4)	172 (95.0)	9 (5.0)	130 (97.0)	4 (3.0)
**Major, n (%)**								
	Clinical		7652 (89.5)	897 (10.5)^b^	4425 (96.7)	151 (3.3)^b^	5098 (96.9)	161 (3.1)^c^
	Basic medical		3002 (87.6)	426 (12.4)	2801 (96.5)	101 (3.5)	2367 (95.9)	100 (4.1)
	Nonmedical		1305 (86.9)	196 (13.1)	936 (94.6)	53 (5.4)	1046 (96.0)	44 (4.0)
**Have sibling(s), n (%)**								
	No		2223 (95.6)	229 (4.4)^b^	1449 (96.9)	46 (3.1)	1501 (96.4)	56 (3.6)
	Yes		9736 (97.3)	1290 (2.7)	6713 (96.3)	259 (3.7)	7010 (96.6)	249 (3.4)
**Year of enrollment, n (%)**								
	2015		1170 (88.7)	149 (11.3)	914 (97.1)	27 (2.9)^c^	645 (97.0)	20(3.0)^c^
	2016		2076 (88.6)	267 (11.4)	1788 (96.0)	75 (4.0)	1348 (96.1)	55 (3.9)
	2017		2633 (87.9)	364 (12.1)	1452 (96.1)	59 (3.9)	1923 (95.5)	90 (4.5)
	2018		2914 (89.0)	360 (11.0)	2103 (97.0)	66 (3.0)	2407 (97.3)	67 (2.7)
	2019		2708 (89.8)	309 (10.2)	1815 (96.4)	68 (3.6)	2135 (96.8)	71 (3.2)
**Intensity of fear, n (%)**								
	0		96 (97.0)	3 (3.0)^a^	180 (98.4)	3 (1.6)^b^	185 (99.5)	1 (0.5)^a^
	1		100 (98.0)	2 (2.0)	223 (98.2)	4 (1.7)	218 (99.5)	1 (0.5)
	2		216 (97.3)	6 (2.7)	382 (97.7)	9 (2.3)	471 (98.5)	7 (1.5)
	3		293 (97.7)	7 (2.3)	446 (97.4)	12 (2.6)	544 (97.5)	14 (2.5)
	4		980 (95.1)	51 (4.9)	1189 (97.1)	35 (2.9)	1278 (97.6)	31 (2.4)
	5		1921 (93.5)	134 (6.5)	1607 (96.6)	55 (3.3)	1758 (96.8)	59 (3.2)
	6		1781 (92.0)	154 (8.0)	1179 (97.0)	37 (3.0)	1295 (96.2)	51 (3.8)
	7		2088 (90.0)	231 (10.0)	1116 (95.0)	59 (5.0)	1138 (96.3)	44 (3.7)
	8		1086 (87.7)	153 (12.3)	513 (96.7)	18 (3.4)	462 (93.7)	31 (6.3)
	9		3398 (81.4)	778 (18.6)	1327 (94.8)	73 (5.2)	1162 (94.6)	66 (5.4)
**Quarantine level, n (%)**								
	Low		1102 (90.6)	114 (9.4)^b^	6073 (96.4)	228 (3.6)	8501 (96.5)	305 (3.5)
	Medium		4885 (89.6)	564 (10.4)	2032 (96.4)	76 (3.6)	10 (100.0)	0 (0)
	High		6272 (88.2)	841 (11.8)	57 (98.3)	1 (1.7)	0 (0)	0 (0)

^a^*P*<.001.

^b^*P*<.01.

^c^*P*<.05.

**Figure 2:**
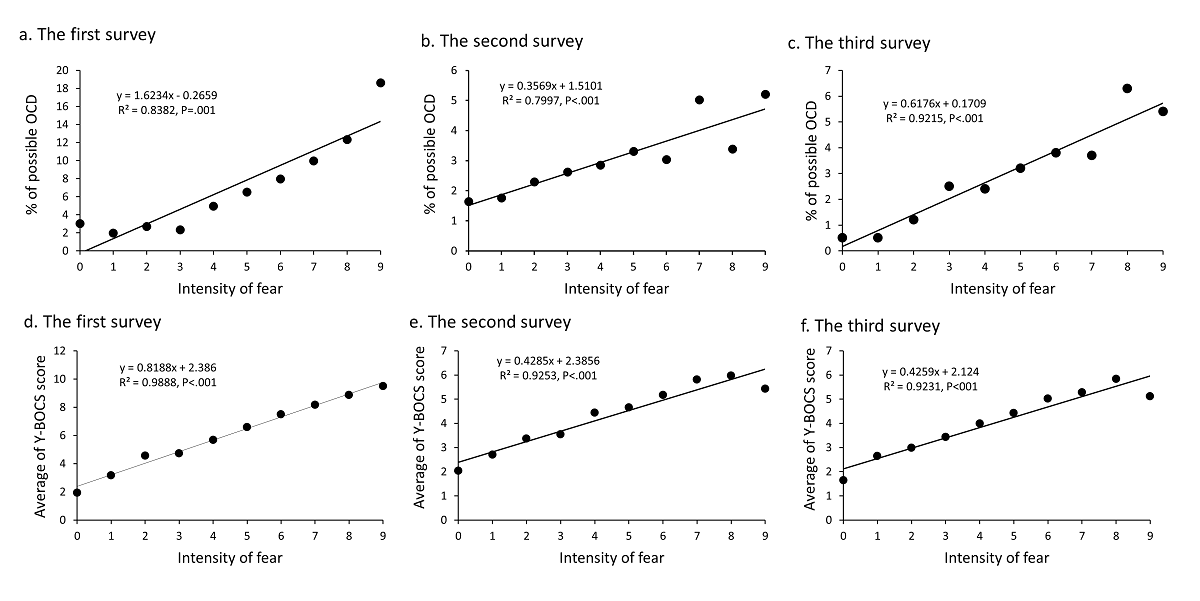
Correlations of the intensity of fear with rates of possible obsessive-compulsive disorder (OCD) and Yale-Brown Obsessive-Compulsive Scale scores.

The regression linear analysis indicated that intensity of fear was significantly correlated to the proportions of possible OCD and the average total scores for the Y-BOCS. The correlation coefficient between intensity of fear and rate of participants with possible OCD was 0.92 for survey 1, 0.89 for survey 2, and 0.96 for survey 3 (*P*<.001 for all). The correlation coefficient between intensity of fear and average Y-BOCS score was 0.99 for survey 1, 0.96 for survey 2, and 0.96 for survey 3 (*P*<.001 for all) (Figure 2).

**Figure 3:**
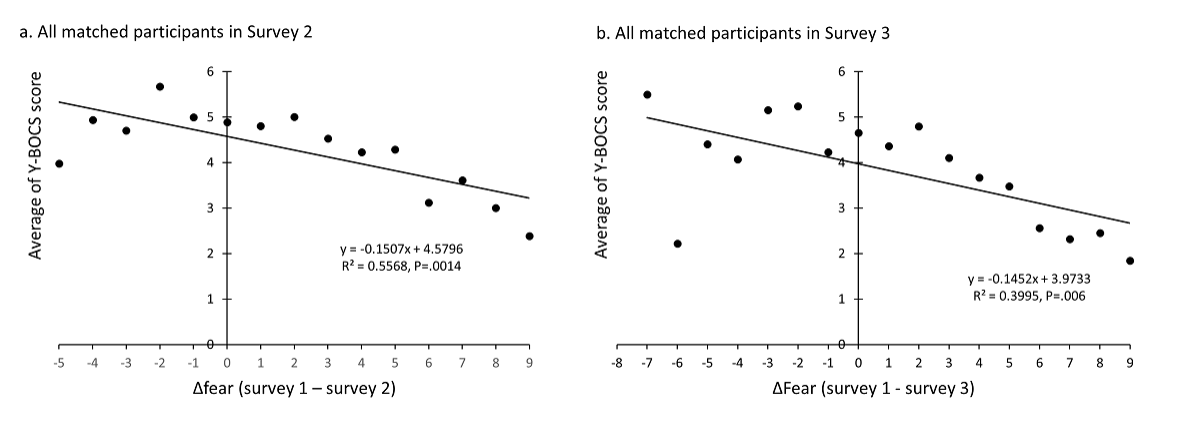
Correlation between changes in the intensity of fear and Yale-Brown Obsessive-Compulsive Scale (Y-BOCS) scores in matched participants.

In the 5827 matched participants from surveys 1 and 2 and the 4006 matched participants from all three surveys, regression analyses indicated that the changes in the intensity of fear (Δfear = fear score from survey 1 – fear score from survey 2 or survey 3) were negatively correlated to the average Y-BOCS score (*P*<.001) (Figure 3).

In order to test the factors that potentially served as predictors for the Y-BOCS score, multiple linear stepwise regressions were conducted, and five variables (intensity of fear, SAS standard score, gender, having sibling[s], and major [1: clinical medicine, 2: basic medical science, and 3: nonmedical major]) entered the equation for all three surveys. Knowledge on COVID-19 entered the equations for surveys 1 and 2 and was negatively correlated to the Y-BOCS score. The quarantine level entered the equation for survey 1 only. Educational level and year of enrollment were excluded from all equations (Table 4). The R^2^ of the regression equation was 0.23 for survey 1, 0.23 for survey 2, and 0.26 for survey 3.

**Table 4:** Multiple linear regression analyses using the Yale-Brown Obsessive-Compulsive Scale score as the dependent variable.

Survey and variables		B	SE	Beta		*t*	*P* value
				Value	95% CI		
**Survey 1**							
	(Constant)	–9.15	0.48	—^a^	–10.09 to –8.22	–19.22	<.001
	SAS^b^ standard score	0.29	0.01	0.40	0.28 to 0.30	52.08	<.001
	Intensity of fear	0.54	0.02	0.19	0.50 to 0.58	24.67	<.001
	Gender	0.50	0.09	0.04	0.32 to 0.68	5.44	<.001
	Having sibling(s)	0.49	0.11	0.03	0.27 to 0.71	4.32	<.001
	Major	0.27	0.06	0.03	0.15 to 0.40	4.29	<.001
	Quarantine level	0.18	0.07	0.02	0.05 to 0.31	2.71	.008
	Knowledge on COVID-19	–0.17	0.09	–0.01	–0.33 to 0.00	–1.96	.03
**Survey 2**							
	(Constant)	–8.37	0.48	—	–9.32 to –7.42	–17.30	<.001
	SAS standard score	0.28	0.01	0.45	0.27 to 0.29	46.35	<.001
	Intensity of fear	0.18	0.02	0.08	0.13 to 0.22	8.10	<.001
	Gender	0.67	0.10	0.06	0.47 to 0.87	6.51	<.001
	Having sibling(s)	0.64	0.13	0.05	0.39 to 0.89	4.97	<.001
	Major	0.21	0.07	0.03	0.07 to 0.35	2.97	.006
	Knowledge on COVID-19	–0.22	0.09	–0.02	–0.40 to –0.05	–2.52	.01
**Survey 3**							
	(Constant)	–9.82	0.36	—	–10.54 to –9.11	–27.02	<.001
	SAS standard score	0.30	0.01	0.48	0.29 to 0.32	51.44	<.001
	Intensity of fear	0.15	0.02	0.07	0.11 to 0.19	7.15	<.001
	Gender	0.38	0.10	0.04	0.19 to 0.58	3.82	<.001
	Having sibling(s)	0.73	0.12	0.05	0.49 to 0.98	5.90	<.001
	Major	0.21	0.07	0.03	0.08 to 0.34	3.19	.006

^a^Not applicable.

^b^SAS: Zung Self-Rating Anxiety Scale.

## Discussion

### Principal Findings

This online prospective cohort study found that the prevalence of possible OCD (11.3%) in survey 1 at the early stage of the COVID-19 pandemic was significantly higher than that in survey 2 (middle stage, 3.6%), and survey 3 (late stage, 3.5%). The Y-BOCS score, anxiety level, quarantine level, and intensity of fear of COVID-19 were significantly lower at surveys 2 and 3 than at survey 1. Compared to those with a lower Y-BOCS score (<16), participants with possible OCD reported a greater intensity of fear and had a higher SAS standard score (*P*<.001). Intensity of fear was positively correlated to the rate of possible OCD and the average total scores for the Y-BOCS in each survey (*P*<.001 for all). Multiple regressions indicated that those with a higher intensity of fear, a higher anxiety level, of male gender, with sibling(s), and majoring in nonmedicine disciplines had a greater chance of having a higher Y-BOCS score across all surveys. In matched survey participants, the Y-BOCS score was negatively correlated to changes in the intensity of fear of COVID-19.

The prevalence of possible OCD in survey 1 was three folds higher than that in surveys 2 and 3, suggesting that possible OCD was induced in the early stage of the COVID-19 pandemic. In addition, the intensity of fear of COVID-19, anxiety level, and quarantine level were significantly higher in survey 1 compared to surveys 2 and 3. Changes in the level of fear were negatively correlated with Y-BOCS score in the follow-up surveys. In each survey, the fear score was strongly correlated to the Y-BOCS score and the rate of possible OCD. The multiple regression analysis showed that both the SAS standard score and intensity of fear significantly contributed to variations in Y-BOCS score. These findings suggest that the intensity of fear of COVID-19 played a role in OCD and that the interactions between fear, anxiety, and pandemic-induced quarantine may be risk factors for an increase in Y-BOCS score. Also, as expected, a high prevalence of possible OCD (11.3%) was observed in survey 1. The COVID-19 pandemic might invoke fear and individuals would manifest OCD-like symptoms when they overacted to this fear. This is an example of the effects of the environment (COVID-19 pandemic) × psychology (fear and/or anxiety) interaction on OCD. Regarding anxiety, a recent longitudinal study on the mental health of the general population during the COVID-19 pandemic that conducted two surveys (1 and 2) at similar intervals to our study did not find a significant reduction in anxiety score in survey 2 [24]. These inconsistent findings may be due to several factors. First, survey 2 in our study was conducted 2 weeks later than that of the longitudinal study. Second, quarantine restrictions had been relaxed in most parts of the country when survey 2 was conducted in this study. Third, we surveyed students in university while the anxiety study surveyed the general population. Fourth, the SAS, which was used in our study, is more sensitive than the Depression, Anxiety and Stress Scale (42 items) used in the other study [25]. Fifth, the participants in this study were taking online courses in surveys 2 and 3, which might have served as a distraction from the pandemic.

In this study, the prevalence of possible OCD decreased from 11.3% at baseline to 3.6% in 5 weeks and remained at 3.5% after 11 weeks. In all three surveys, the participants were at home; hence, the living environment did not vary significantly. At the time of survey 2, participants had been taking online courses at home for 2 weeks, and the pandemic was partially under control. At the time of survey 3, the COVID-19 pandemic was under control; the quarantine level was lowered further, and the participants continued taking online courses at home. The intervals between the three surveys were 5-6 weeks. Therefore, compared to survey 1, the changed environmental factors at surveys 2 and 3 mainly included the status of the pandemic, level of quarantine, and online courses, while the changed psychological factors at surveys 2 and 3 included the intensity of fear of COVID-19, the decreased anxiety level, and the provision of more knowledge on COVID-19. Statistical analyses indicated that the quarantine level and knowledge on COVID-19 were not correlated to Y-BOCS score in survey 3, while knowledge on COVID-19 was negatively correlated to Y-BOCS score and explained less than 1% of the variation seen in the scores. The decreases in intensity of fear and anxiety level may be related to reductions in quarantine level due to declines in new case reports and dissemination of more knowledge on COVID-19 and may also be related to the interaction between those factors and time. In addition, taking online courses could be considered as an intervention to reduce fear of COVID-19 and anxiety, since more time spent on coursework is less time spent on activities that may instigate fear of COVID-19 and COVID-19–related anxiety. Reduction in the intensity of fear and anxiety was correlated to Y-BOCS score, leading to lower rates of possible OCD. Taking online courses is similar to strategies used in OCD treatment (eg, cognitive-coping therapy) [10,11]. That, subsequently, might be related to the lower rate of possible OCD in a relatively short period of time.

Not all participants with fear of COVID-19 were categorized as possible OCD based on Y-BOCS score, although our findings indicate that a higher intensity of fear was related to a higher prevalence of possible OCD. Previously, we investigated the relationship between the fear of negative events and OCD on patients, and found that for most patients with OCD a fear of negative events contributed to their symptoms [10-12].

The findings in this study introduced a new perspective to understanding the relationship between fear of negative events and OCD in the general population. First, it is not unusual that most participants in this study reported a certain intensity of fear related to the COVID-19 pandemic. Second, the attitude, evaluation, and cognition of this fear may affect their response to it. Third, when fear is excessive and disproportionate to the situation, it could lead to the development of an anxiety disorder [26,27]. Those who took the fear seriously and overreacted to it were more likely to be categorized as possible OCD. Fourth, the environment × psychology interaction could be a risk factor for some people and a resilience factor for others due to value-system differences.

We noted that 3 out of 1519 cases of possible OCDs in survey 1, 3 out of 305 cases of possible OCDs in survey 2, and 3 out of 305 cases of possible OCDs in survey 3 reported that their intensity of fear was zero. However, they reported fear of bodily waste/secretions, dirt or germs, infectious illnesses, and environmental contamination. In the matched samples, no participant who reported zero for intensity of fear had a Y-BOCS score ≥16.

In addition, the participants who reported having sibling(s) were more likely to be categorized as possible OCD than those who had no sibling(s). In China, an only child easily becomes the family’s center of attention and gets care from not only parents but also grandparents, even throughout early adulthood. Only children have closer parent-child relationships, which is probably related to being dependent upon others and having relatively few familial responsibilities [28]. On the other hand, those with siblings generally take on more responsibilities, such as caring for siblings or assisting in family affairs. Participants with sibling(s) may be more responsible and thus overreact to COVID-19 during the pandemic and be involved in transient possible OCD.

Our findings demonstrated that the prevalence of possible OCD in surveys 2 and 3 was 3.6% and 3.5%, respectively. Additionally, the prevalence of possible OCD in male participants at all timepoints was higher (12.5%, 4.4%, and 4.5% for surveys 1, 2, and 3, respectively) compared to females (10.6%, 2.7%, and 2.9% for surveys 1, 2, and 3, respectively). The findings suggested that the male students in the present study seem to have a higher prevalence of OCD than the general population [5]. Previously, Torres et al [29] found that 3.8% of Brazilian medical students had a possible case of OCD based on the Obsessive-Compulsive Inventory–Revised. Using the OCD subsection of the Clinical Interview Schedule–Revised as a self-administered questionnaire, Jaisoorya et al [30] reported the point prevalence of OCD in Indian college students as 3.3% (males: 3.5%; females: 3.2%). Yoldascan et al [31] reported that, in Turkish university students, the prevalence of OCD is 4.2%, and OCD was significantly associated with the male gender. These findings, along with ours, suggest that the prevalence of OCD among university students is similar to or higher than the general population across different cultures.

Our survey results indicated that the prevalence of possible OCD was positively correlated to the participant’s academic discipline. Basic medical students (ie, those not involved in clinical practice) had a higher prevalence of possible OCD than medical students, but a lower prevalence of possible OCD than nonmedical students. Additionally, students in their third and fourth years had a higher prevalence of possible OCD than first-, second-, and fifth-year students. The findings suggested that the knowledge that students obtained in their academic majors may play a role in the “onset” of possible OCD, possibly by affecting one’s cognition and appraisal of fear of COVID-19. Cognitive-behavior therapy might be useful to treat possible OCD and anxiety symptoms [32].

### Limitations

Although this study included a pragmatic design and a large sample size, there are several limitations that need to be addressed. First, individuals with possible OCD were defined only according to the Y-BOCS score and were not verified via face-to-face interview, which might be related to the higher prevalence of possible OCD in this study compared to the general population. Second, we did not collect any biological samples and therefore could not analyze the relationship between OCD and factors such as genetics and/or the expression of certain proteins. Third, all participants were university students aged 17 to 50 years. Therefore, caution is advised if using the findings to infer patterns in the general population.

### Conclusions

Our findings indicate that an environment × psychology interaction might be involved in the onset of OCD and that a fear of negative events should be considered as a target of interventions for mental health and well-being in both stressful situations and in clinical practice.

## Abbreviations

ANOVA: analysis of variance
OCD: obsessive-compulsive disorder
OR: odds ratio
SAS: Zung Self-Rating Anxiety Scale
XXMU: Xinxiang Medical University
Y-BOCS: Yale-Brown Obsessive-Compulsive Scale
